# Electronic Patient Portal Use Among People Living With HIV

**DOI:** 10.2196/47740

**Published:** 2023-11-21

**Authors:** André Hall, Samantha Devlin, Joshua Won, Jessica Schmitt, Jessica P Ridgway

**Affiliations:** 1 Section of Infectious Diseases Department of Medicine University of Chicago Chicago, IL United States

**Keywords:** HIV, patient portal, EMR, electronic medical record, health record, health records, medical record, medical records, patient portals, infectious, EHR, electronic health record, use, adoption, quality improvement, engagement, satisfaction

## Introduction

Electronic patient portals are secure websites or web-based applications that give patients access to their electronic medical records and allow for secure messaging between patients and providers. Patient portals have been associated with greater patient satisfaction and engagement in care [1-3] and fewer no-show appointments [4].

For people living with HIV (PLWH), engagement in health care is essential for improving individual health and limiting the transmission of HIV. While a prior study demonstrated that PLWH have an interest in using patient portals [5], little is known about portal engagement among PLWH. The purpose of this project was to survey PLWH to understand facilitators and barriers to portal use and implement a brief intervention to enroll PLWH in the electronic patient portal.

## Methods

### Overview

From February to April 2022, we surveyed adult PLWH at the University of Chicago Medicine HIV care clinic regarding their use and perceptions of MyChart, the patient portal used at the University of Chicago Medicine. MyChart allows patients to message providers, request prescription refills, and view lab results, among other functionalities [6]. PLWH who are 18 years or older were eligible to participate. We approached patients during regularly scheduled appointments with their HIV clinicians. For patients who agreed to be surveyed, we collected demographics and asked questions regarding their MyChart use, barriers to MyChart, interest in MyChart, and reasons why they used or did not use MyChart (Multimedia Appendix 1).

For patients who were not initially enrolled in MyChart, we offered to enroll them by providing a unique activation code, assisting them with downloading the smartphone app, and providing a tutorial for MyChart features.

We compared the results for patients enrolled versus those not enrolled in MyChart using chi-square and Fisher exact test for categorical variables and *t* tests for continuous variables. We also analyzed open-ended responses qualitatively to identify common themes for reasons why patients chose to use or not to use MyChart.

### Ethical Considerations

This project underwent a formal review and received a quality improvement determination according to the University of Chicago’s institutional policy. Thus, this initiative was not considered human subjects research and was not reviewed by the institutional review board.

## Results

A total of 66 patients were surveyed (Table 1), and 21 (32%) were not enrolled in MyChart. Of these, 13 (62%) opted to newly enroll in MyChart. Of these 13 patients, 6 (46%) cited being unaware of MyChart as the reason they were previously not enrolled. Of the 21 patients not enrolled, 8 (38%) declined enrollment, with most (n=7, 88%) citing “technological mistrust” and “dislike of the internet” as reasons.

Most of the 66 patients (n=45, 68%) were already enrolled in MyChart. These patients predominantly used MyChart for viewing lab results, messaging providers, confirming appointment times, and requesting prescription refills.

**Table 1:** Participant demographics (N=66).

		Total participants (N=66)	Enrolled in MyChart (baseline; n=45)	Not enrolled in MyChart (baseline; n=21)	*P* value	
**Gender identity, n (%)**						.96
	Men	50 (76)	34 (76)	16 (76)		
	Women	16 (24)	11 (24)	5 (24)		
**Race, n (%)**						.01
	Black	55 (83)	34 (76)	21 (100)		
	Other	11 (17)	11 (24)	0 (0)		
**Insurance status, n (%)**						.01
	Private	20 (30)	18 (40)	2 (10)		
	Medicaid	23 (35)	11 (24)	12 (57)		
	Medicare	23 (35)	16 (36)	7 (33)		
Age (years), median (range)		49 (26-75)	48 (26-73)	53 (27-75)	.17	

## Discussion

Most of the PLWH who were surveyed were enrolled in MyChart at baseline. Patients indicated they enjoyed the platform’s ability to view lab results, message providers, confirm appointment times, and request prescription refills. Our findings support the notion that patient portals are beneficial tools for promoting engagement in care for PLWH and patient-provider communication.

Among PLWH who were not using the portal, a brief intervention resulted in the enrollment of the majority of these individuals. Patients who decided to enroll in MyChart reported “being unaware” of MyChart as the primary reason for not enrolling previously. These results are similar to a prior study among PLWH that identified a lack of information and the need for more education as common reasons for low enrollment in patient portals [7]. However, we also found that several participants reported “dislike of the internet” and “technological mistrust” as important reasons for not enrolling. Therefore, ways to raise awareness of and promote trust in patient portals among PLWH should be explored.

While our sample size was too small to thoroughly investigate issues of equity regarding portal access, equity may impact portal access. Our patients with Medicare or Medicaid were less likely to be enrolled in MyChart than patients with private insurance, and Black patients were less likely to use MyChart than patients of other races. We did not measure income or other social determinants of health, which may play a more important role in equity than race. More research is needed to understand these disparities, and portal enrollment efforts should focus on increasing enrollment among these groups.

The use of MyChart among PLWH was examined only at one point in time. Future studies examining the active portal use of MyChart among enrolled patients, as well as its relation to HIV care continuum outcomes among PLWH, are also warranted.

## Appendix

Multimedia Appendix 1 Investigator-developed questionnaire.

## Abbreviations

PLWH: people living with HIV
