# Supplementary material for: Electronic Patient Portal Use Among People Living With HIV
Source: J Med Internet Res. 2023 Nov 21;25:e47740. doi: 10.2196/47740 (PMC10698658; doi:10.2196/47740)
Supplement: Multimedia Appendix 1 [file jmir_v25i1e47740_app1.pdf]

Investigator-Developed Questionnaire

Medical Record Number ____________________

Patient MRN from EPIC ____________________

Date of interview ____________________

Demographics

First Name ____________________

Last Name ____________________

Date of birth ____________________
(MM-DD-YYYY)

Age (years) ____________________

Sex at Birth
- ◯ Male
- ◯ Female

Gender
- ◯ Male
- ◯ Female
- ◯ Non-binary

Race
- ◯ American Indian/Alaska Native
- ◯ Asian
- ◯ Black or African American
- ◯ Native Hawaiian or Other Pacific Islander
- ◯ White
- ◯ More Than One Race
- ◯ Patient Declined
- ◯ Unknown / Not Reported

Ethnicity
- ◯ Hispanic or Latino
- ◯ Not Hispanic or Latino
- ◯ Declined
- ◯ Unknown / Not Reported

Insurance Provider ____________________
(Name of insurance)

Financial Class	◯ Private Employer<br>◯ Private Individual<br>◯ Medicare<br>◯ Medicaid<br>◯ Other<br>◯ Uninsured
MyChart status (EPIC variable)	◯ Activated<br>◯ Inactivated<br>◯ Pending
Does the patient report using MyChart?	◯ Yes<br>◯ No
If not using MyChart, why not?	__________________________________
Would you like to sign up?	◯ Yes<br>◯ No
Date of MyChart activation	__________________________<br>((MM/DD/YYYY))
Date of last login?	__________________________
Does the patient use MyChart regularly?	◯ Yes<br>◯ No
What does the patient mainly use MyChart for?	__________________________________
What technology does the patient use to access MyChart?	☐ Computer<br>☐ Smartphone
Would the patient like to use MyChart?	◯ Yes<br>◯ No
Would the patient like you to show them how to use MyChart (either on their phone and/or computer)?	◯ Yes<br>◯ No
In what ways did you help the patient with MyChart?	☐ Enrolled patient in MyChart<br>☐ Downloaded MyChart app on phone<br>☐ Provided demonstration - scheduling<br>☐ Provided demonstration - messaging provider<br>☐ Other
Other:	__________________________
Should we follow up in any way with this patient?	◯ Yes<br>◯ No

Notes
